# Remimazolam protects the liver from ischemia-reperfusion injury by inhibiting the MAPK/ERK pathway

**DOI:** 10.1186/s12871-024-02641-3

**Published:** 2024-07-25

**Authors:** Yanhua Shi, Housheng Deng, Zhiming Zhang, Xiaoling Zhu, Zhiqin Zeng

**Affiliations:** Department of Anesthesiology, Chenzhou First People’s Hospital, No.102 Luojiajing, Beihu District, Chenzhou City, 423000 Hunan Province People’s Republic of China

**Keywords:** Remimazolam, Ischemia-reperfusion, Liver injury, MAPK/ERK pathway

## Abstract

**Background:**

Ischemia-reperfusion (I/R) injury is a major factor in liver damage following hepatic resection and liver transplantation, with anesthetics demonstrating the ability to shield organs from this type of injury.

**Methods:**

Hypoxia-reoxygenation (H/R) was used to create in vitro I/R hepatocyte cell injury models. The CCK-8 assay, flow cytometer, LDH assay, and ELSIA were utilized to assess hepatocyte injury. The in vivo I/R injury rat model was then built. HE and TUNEL staining were used to assess liver tissue damage. Western-blot was applied to assess the activation of the MAPK/ERK pathway.

**Results:**

Remimazolam (RMZL) remarkably improved cell viability and decreased apoptosis in H/R-induced hepatocyte injury. RMZL reduced the release of H/R-induced inflammatory mediators (TNF-α and IL-6) as well as LDH levels. We also discovered that RMZL inhibited p38 and ERK1/2 phosphorylation in vivo and in vitro. The stimulation of MAPK/ERK, on the other hand, abolished RMZL’s anti-inflammation effects in H/R-induced hepatocyte injury. Furthermore, RMZL reduced liver tissue injury in I/R rats.

**Conclusion:**

RMZL prevented hepatic I/R damage by inhibiting MAPK/ERK signaling.

**Supplementary Information:**

The online version contains supplementary material available at 10.1186/s12871-024-02641-3.

## Introduction

Ischemia-reperfusion (I/R) injury is the pathological process of injury worsening caused by blood flow being restored to the tissue after ischemia for a period of time, which typically occurs after surgery and organ transplantation [1, 2]. I/R damage frequently affects many organs, is followed by significant inflammatory responses, and results in cell injury and death [3–5]. I/R injury is a major source of liver damage during surgical procedures including liver transplantation and hepatic resection, and it is the main contributory factor in graft failure following transplantation [6, 7]. Finding ways to reduce I/R injury can help alleviate liver damage after liver surgery.

Anesthetics are used to provide pain relief and sedation during surgical procedures. Recent investigations has indicated that anesthetics also possess anti-inflammatory properties [8]. Studies have shown that anesthetics like sevoflurane, propofol, and isoflurane have protective effects on the heart, brain, and kidney of patients undergoing I/R injury [9–11]. Remimazolam (RMZL) was additionally demonstrated to protect rats from cerebral I/R injury [12]. RMZL, a new benzodiazepine for procedural sedation and general anesthesia, was found to be a safer and more effective choice for procedural sedation [13]. In the case of liver and kidney damage, RMZL was a safer sedative than midazolam [14]. However, the effects of RMZL on hepatic I/R injury have not been studied, and whether it plays a protective role in hepatic I/R injury is unknown.

The extracellular signal-regulated kinase (ERK) is a part of the mitogen-activated protein kinase (MAPK) family and is involved in regulating various cellular functions such as cell proliferation, differentiation, survival, death, and transformation [15]. The MAPK/ERK signaling was implicated in the regulation of I/R injury [16], and inhibiting this pathway has been found to reduce early proinflammatory and stress-response gene expression, effectively lessening hepatic I/R injury after liver transplantation [17]. Research has shown that RMZL can decrease the severity of sepsis-related acute liver damage by blocking macrophage p38 activation [18]. However, it is unclear whether RMZL can also play a role in hepatic I/R injury via the MAPK/ERK pathway. Therefore, we studied the effects of RMZL on the MAPK/ERK pathway in hepatic I/R injury utilizing animal and cell experiments.

## Methods

### Cell culture and treatment

The rat normal liver cell line (Buffalo rat liver-3 A, BRL-3 A) was obtained from ATCC (VA, USA) and grown in DMEM-F12 media supplemented with insulin-transferrin-selenium solution (Gibco, CA, USA), 40 ng/mL dexamethasone (Sigma-Aldrich, Merck, Germany), and 10% FBS (Gibco). The hypoxia-reoxygenation (H/R) cell model was used to simulate I/R injury in vitro. BRL-3 A cells were exposed to hypoxic conditions (5% CO_2_, 94% N_2_ and 1% O_2_) for 12 h before being cultured in normal conditions (5% CO_2_ and 95% air) for 4 h. In addition, BRL-3 A cells were subjected to a constant concentration (10, 50, 100 µM) of RMZL (PAION UK Ltd, Cambridge, UK) after H/R treatment.

### Cell counting kit (CCK)-8

The viability of BRL-3 A cells was determined through the use of a CCK-8 test kit (Beyotime Biotechnology, Shanghai, China). Cells (1 × 10^4^ cells/well) were put in 96-well culture plates and left to grow for 24 h. The cells were then treated differently. After that, each well received 20 µL of CCK-8 solution and was incubated for 1 h. The absorbance of each well was measured at 450 nm.

### Apoptosis detection

For apoptosis detection, an Annexin V-FITC/PI Apoptosis Detection Kit (Beyotime) was employed. The cells were stained for 15 min in the dark with 5 µL of FITC-conjugated antibodies and 5·µL·of propidium iodide (PI). A flow cytometer (BD Biosciences, NJ, USA) was used to determine the level of cell apoptosis. The data were analyzed with the FlowJo program (Tree Star, Ashland, OR).

### Lactate dehydrogenase (LDH) and inflammatory factor levels were determined

The LDH level in BRL-3 A cells was determined using a commercial kit (Abcam, Cambridge, UK) in line with the instructions of the product creator. The concentration of LDH in each well was detected at a wavelength of 450 nm. TNF-α and IL-6 levels were performed strictly in accordance with the directions of the enzyme-linked immunosorbent assay kits (Beyotime).

### Immunofluorescence (IF) analysis

The BRL-3 A cells were cultivated on coverslips in 24-well plates for 12 h at a density of 1 × 10^4^ cells/well. Following various treatments, cells were fixed for 15 min in 4% paraformaldehyde. They were then permeabilized with 1% Triton X-100 (Sigma Aldrich, Darmstadt, Germany) and blocked with 3% BSA (Solarbio, Beijing, China). After that, cells were incubated at 4°C overnight with the p38 antibody (#9212, 1:50, Cell Signaling Technology, USA). The cells were then treated with Alexa Fluor 488 Conjugate secondary antibodies (#4412, 1:2000, Cell Signaling Technology) for 1 h at room temperature and DAPI (Roche) for 30 min. Cells were mounted and analyzed using a confocal laser scanning microscope (Olympus, Tokyo, Japan). ImageJ software was used to process and analyze photographs.

### Western blotting

Cells and tissues were lysed in RIPA buffer (Solarbio) for 30 min at 4 °C with protease inhibitors (Roche Diagnostics, Mannheim, Germany) and phosphatase inhibitors (PMSF from Beyotime). A SDS-PAGE method was used to separate the proteins, which were then transferred to a PVDF membrane (Millipore, Bedford, MA, USA) and blocked for 2 h with 5% skimmed milk. Following that, the membrane was incubated at 4 °C with primary antibodies against p38 (#9212, 1:1000), p-p38 (#4511, 1:1000), ERK1/2 (#4695, 1:1000), and p-ERK1/2 (#4370, 1:2000). As an internal reference, GAPDH (#5174, 1:1000) was employed. The membranes were then treated for 1 h at room temperature with the HRP-labeled second antibody (#7074, 1:3000). All of the antibodies were purchased from Cell Signaling Technology. Enhanced chemiluminescence reagent (ECL, Amersham) were applied, and membranes were observed using the Bio-rad gel imager (Bio-Rad, California, USA). The Image Lab program was used to assess the intensity of the bands.

### Animal and hepatic I/R injury model

A total of twenty-four male SPF grade Sprague-Dawley rats (190–210 g) were provided by Sun Yat-Sen University [SCXK (Guangdong) 2021-0029]. Rats were raised in a room at 20 °C and 60% humidity, with daily access to water and food. The rats were maintained in a 12 h light/dark cycle. After one week of feeding, the rats were randomly assigned into four groups: Control group, I/R group, I/R + RMZL group and RMZL group.

Animal experiments were approved by Chenzhou No.1 People’s Hospital’s ethics committee and reported in accordance with ARRIVE guidelines. An intraperitoneal dose of 50 mg/kg pentobarbital (B5646-50 mg, ApexBio, USA) was used to anesthetize the rats. Hepatic I/R surgical procedures were referred to Liu et al. [19]. In brief, rats were placed on the surgical table in the supine posture. The livers of the rats were exposed when the abdomens of the rats were opened. The hepatic pedicle was then clamped with a noninvasive microvascular clamp until the left and center lobes became white, at which point the vascular clips were removed and the belly sutured. The rats were given free access to food and water while recovering. RMZL (8 mg/kg) was subcutaneously infused 30 min following hepatic I/R surgery. The RMZL group consisted of rats who only received RMZL. After 24 h of reperfusion, all of the animals were euthanized, and their livers were removed for tissue section staining and protein extraction.

### Hematoxylin-eosin (HE) staining

The liver tissues were fixed, embedded, and cut into 5 μm slices. The sections were then baked, dewaxed with xylene, and hydrated with graded alcohol. Following hematoxylin and eosin staining, the slices were dehydrated, cleared and sealed before being examined under an optical microscope.

### TdT-mediated dUTP Nick-End labeling (TUNEL) assay

According to the directions of commercially available TUNEL kits (Beyotime), TUNEL staining was used to identify apoptosis in liver tissues. After dewaxing and hydrating the paraffin liver slices, the liver sections were digested for 20 min at room temperature with proteinase K at concentration of 20 µg/mL. The liver tissue sections were then washed and incubated for 1 h at 37 °C with biotinylated labeled TUNEL reaction solution. Following that, a diaminobenzidine tetrahydrochloride substrate (Invitrogen) and hematoxylin stain were applied. A microscope (Olympus, Japan) was used to examine the slices. Random calculations were made to determine the percentage of TUNEL-positive cells in five non-repetitive fields.

### Statistical analysis

Results were reported as means ± SD. Figures and analyses were generated using the GraphPad Prism 8 software. The experimental analysis was carried out in triplicate, with a minimum of three independent experiments. Student’s t-test was used to compare two groups, and one-way ANOVA followed by the Tukey test was applied to compare multiple groups. *p* < 0.05 was considered statistically significant.

## Results

### RMZL has protective effects on H/R treated hepatocytes injury

**Fig. 1:**
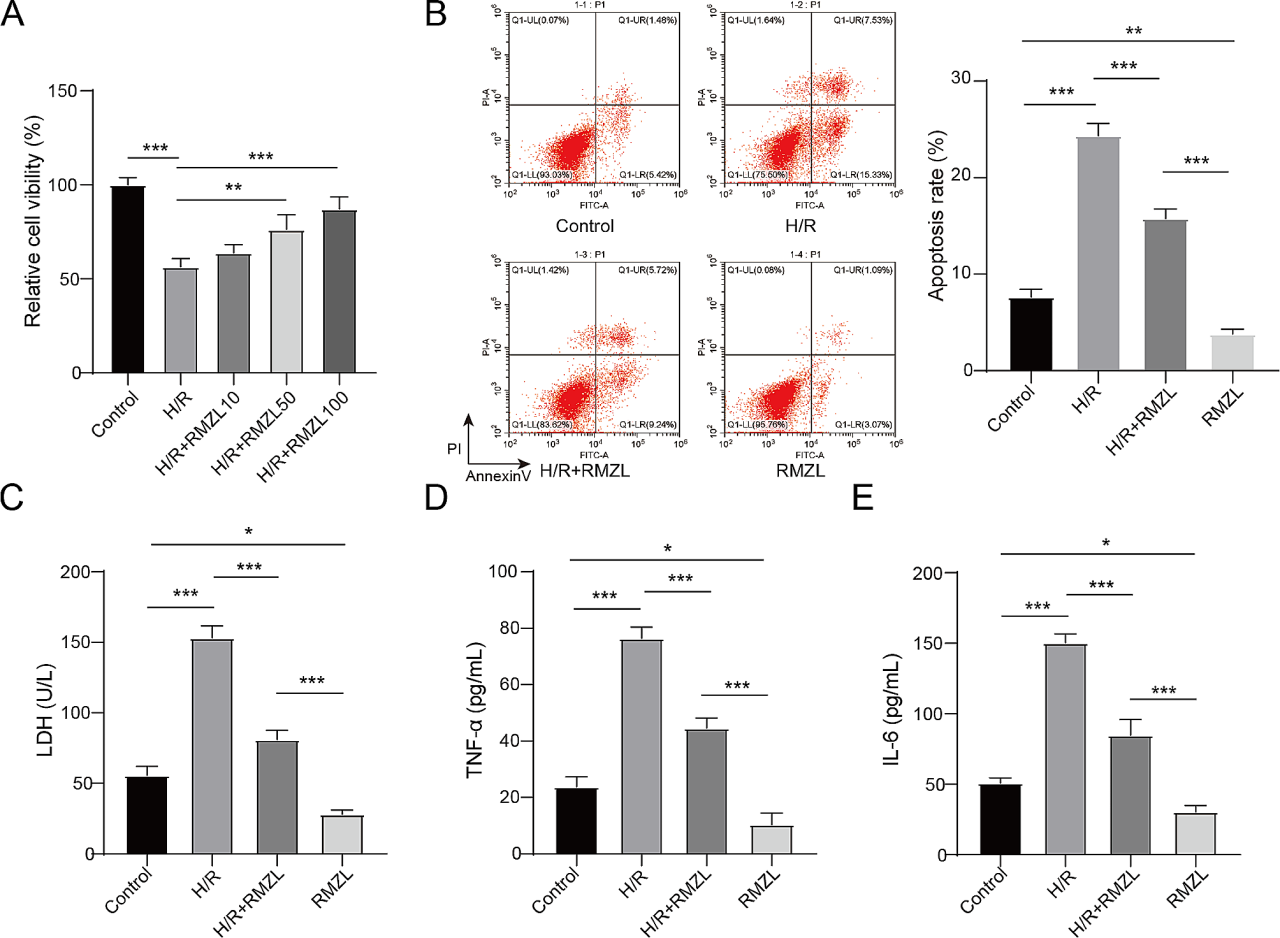
RMZL has protective effects on H/R-induced hepatocytes injury. (**A**) The relative cell viability of BRL-3 A in each group was evaluate by CCK-8 assay. (**B**) The cell apoptosis rate of BRL-3 A was detected by the flow cytometer. (**C**) The LDH levels were determined via the commercial kit. (**D-E**) The levels of TNF-α and IL-6 were measured by ELISA. *P < 0.05, **P < 0.01, ***P < 0.001

First, we looked into whether RMZL could protect H/R-induced hepatocytes injury. Based on the findings in Fig. 1A, the survival rate of BRL-3 A cells decreased following H/R treatment. However, the cell survival rate notably rose in a dose-dependent manner with RMZL treatments at concentrations of 50 µM and 100 µM. Therefore, a concentration of 100 µM was chosen for subsequent trials. In comparison to the control group, RMZL declined the rate of cell apoptosis in hepatocytes. In addition, RMZL dramatically reduced H/R-induced hepatocyte apoptosis (Fig. 1B). Furthermore, RMZL suppressed the expression of LDH, TNF-α, and IL-6 in BRL-3 A and decreased the release of these factors in H/R-induced hepatocytes (Fig. 1C-E). Above all, RMZL protected hepatocytes form H/R-induced damage.

### RMZL inhibited the activation of the MAPK/ERK pathway in H/R treated hepatocyte

**Fig. 2:**
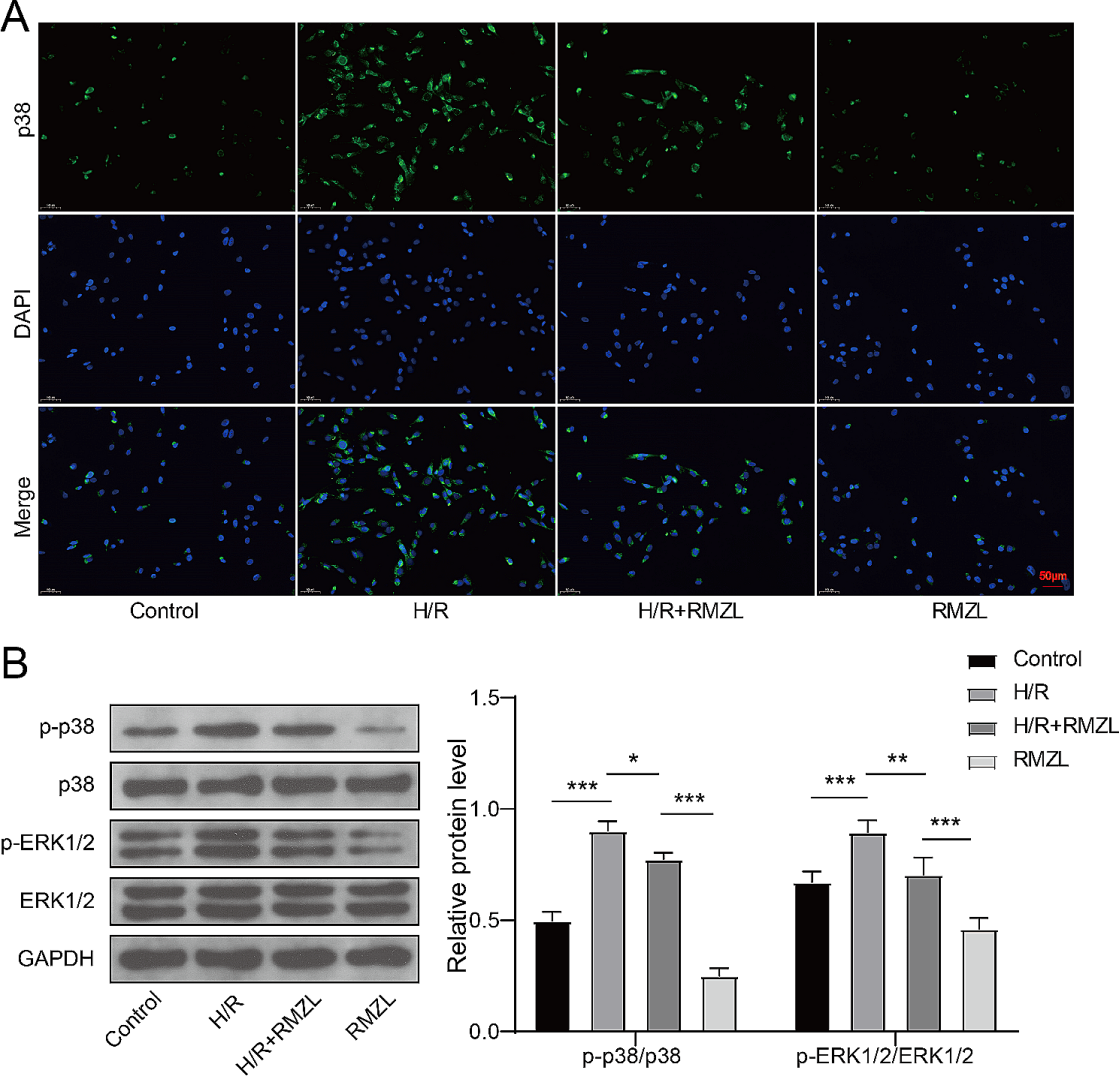
RMZL inhibited the activation of the MAPK/ERK pathway H/R-induced hepatocytes. (**A**) The expression level of p38 was monitored by IF. (**B**) The protein levels of p38, p-p38, ERK1/2, and p-ERK1/2 were detected by western blotting. *P < 0.05, **P < 0.01, ***P < 0.001

Given that MAPK/ERK signaling has been associated with the regulation of I/R damage [17], we examined whether RMZL affects the MAPK/ERK signaling status in hepatocytes. Compared to the control group, RMZL reduced p38 expression in BRL-3 A cells and halted the elevation of p38 levels following H/R treatment (Fig. 2A). Additionally, the levels of p-p38 and p-ERK1/2 were increased in H/R treated hepatocytes, but RMZL prevented the phosphorylation of p38 and ERK1/2 from rising (Fig. 2B-C). This demonstrated that RMZL inhibited H/R-induced activation of the MAPK/ERK pathway in hepatocytes.

### The anti-inflammatory effects of RMZL in H/R-induced hepatocytes was achieved through inhibiting the MAPK/ERK pathway

**Fig. 3:**
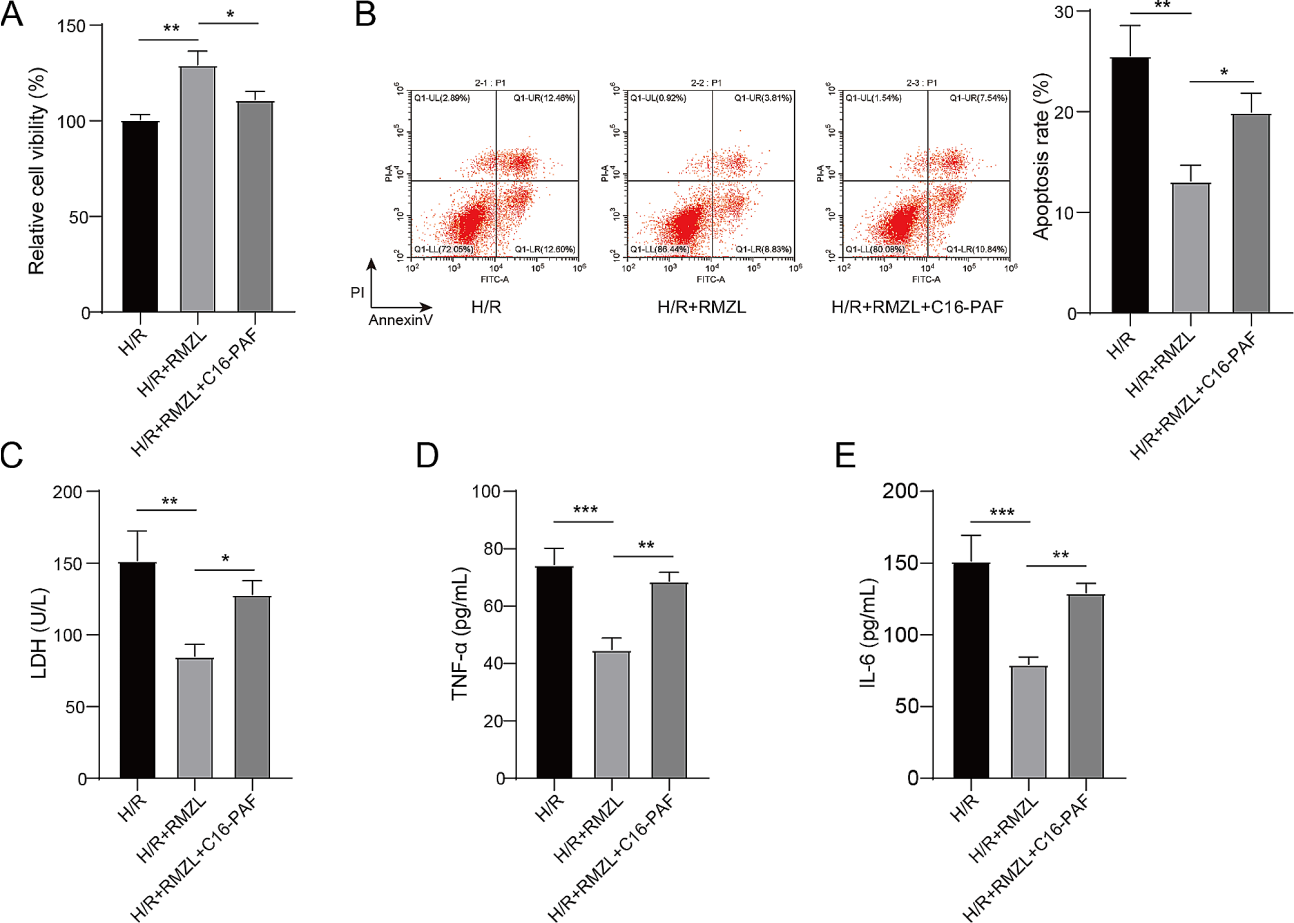
The anti-inflammatory effects of RMZL in H/R-induced hepatocytes was achieved through inhibiting the MAPK/ERK pathway. (**A**) The relative cell viability of BRL-3 A in each group was evaluate by CCK-8 assay. (**B**) The cell apoptosis rate was detected by the flow cytometer. (**C**) The LDH levels were determined via the commercial kit. (**D-E**) The levels of TNF-α and IL-6 were measured by ELISA. *P < 0.05, **P < 0.01, ***P < 0.001

C16-PAF, which activates the MAPK/ERK signaling pathway, was then introduced to confirm whether RMZL could mitigate hepatocyte injury induced by H/R by blocking the MAPK/ERK pathway. The addition of C16-PAF reversed the increased cell viability observed in the RMZL + H/R group (Fig. 3A), as well as the protective effect of RMZL on cell apoptosis in H/R-treated hepatocytes (Fig. 3B). Furthermore, C16-PAF significantly diminished the effectiveness of RMZL in reducing the production and release of LDH, TNF-α, and IL-6 in H/R-induced hepatocytes (Fig. 3C-E). Thus, RMZL protects hepatocytes from H/R-induced damage by inhibiting MAPK/ERK activation.

### RMZL inhibited the MAPK/ERK pathway to reduce liver damage in rats subjected to I/R injury

**Fig. 4:**
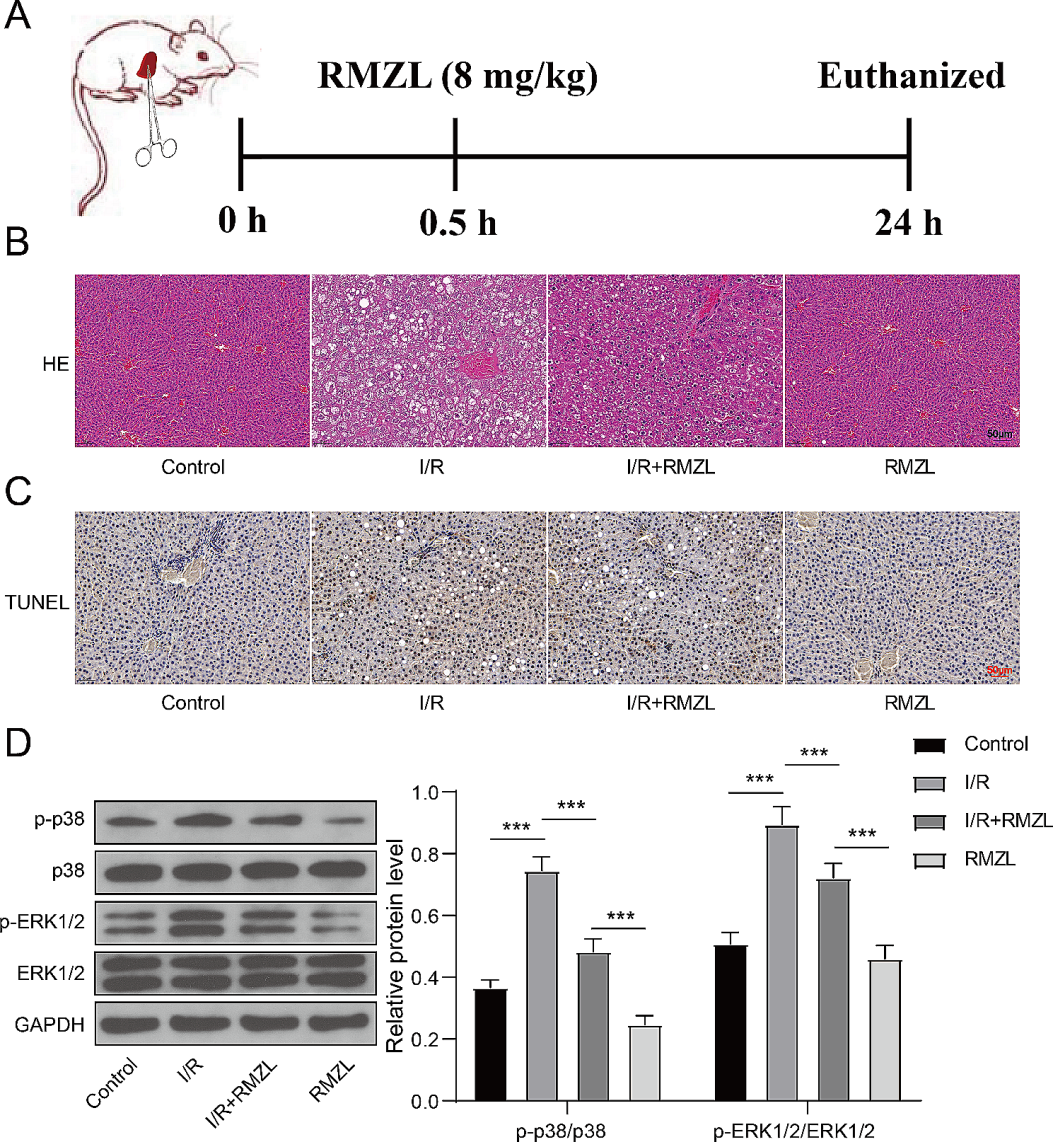
RMZL inhibited the MAPK/ERK pathway to reduce liver damage in rats subjected to I/R injury. (**A**) The scheme of animal experiments. (**B**) The level of damage in the liver tissues was assessed by HE staining. (**C**) Cell apoptosis in liver tissue was counted using TUNEL staining. (**D**) Protein levels of p38, p-p38, ERK1/2, and p-ERK1/2 in liver tissues were determined using western blotting. *P < 0.05, **P < 0.01, ***P < 0.001

The study also demonstrated the protective impact of RMZL on liver damage induced by I/R in vivo**(**Fig. 4A**)**. I/R led to liver tissue damage and cell death, but RMZL treatment alleviated these effects (Fig. 4B-C). In contrast to the control group, the levels of p-p38 and p-ERK1/2 were elevated in liver tissues of I/R rats, while RMZL therapy dramatically lowered the levels of these proteins in liver tissues (Fig. 4D). In conclusion, RMZL supressed the MAPK/ERK pathway to alleviate liver damage in vivo.

## Discussion

Current I/R therapeutic techniques generally target on lowering ROS levels, complement levels, directly or indirectly diminishing inflammatory mediators, preventing immune cell activation, and polarization of macrophages [2]. The liver is sensitive to I/R-induced inflammation. The inflammation caused in I/R hepatic cells not only yields acute-phase hepatic damage, but it also contributes to cancer recurrence and fibrogenesis in the long term [20]. Thus, interventions that can minimize the short- and long-term impacts of I/R-induced hepatic inflammation are efficient and promising. We discovered in this work that RMZL effectively mitigated I/R-induced hepatocyte damage by suppressing MAPK/ERK pathway.

The anti-inflammatory protective effect of anesthetics on I/R has been proven. Propofol has been demonstrated to inhibit TNF-α, IL-6, and CXCL-10 expression in I/R-induced kidney injury [21]. In myocardial I/R injury mice, etomidate reduced inflammatory factor release, which helped to ameliorate cardiac dysfunction [22]. Sevoflurane protected against cerebral I/R by blocking the TLR4/NF-κB signaling pathway, which has been associated to inflammatory and immunological responses [23]. In this investigation, we discovered that RMZL decreased the expression of inflammatory markers in hepatocytes and reduced hepatocyte damage in I/R. Furthermore, MAPK/ERK pathway was activated in I/R-induced hepatocyte damage, but RMZL prevented MAPK/ERK pathway activation in I/R-induced hepatocyte damage.

Upregulation of MAPK activation has been found to operate as a triggering mechanism in hepatic dysfunction and produce liver damage in I/R rats, and the level of phosphorylation ERK1/2 was significantly raised in I/R rats after a brief period of reperfusion and lasted for a long time [24]. The suppression of MAPK/ERK pathway activation decreased the levels of proinflammatory mediators and reduced transplant-induced hepatic I/R damage [17]. A recovery experiment was then used to demonstrate the link between RMZL and MAPK/ERK in hepatic I/R damage. The addition of MAPK/ERK agonists reduced RMZL’s therapeutic impact on H/R hepatocyte injury. Furthermore, the addition of agonists enhanced the levels of inflammatory cytokines and cell damage. As a result, we came to the conclusion that RMZL reduced H/R-induced hepatocyte inflammatory injury via blocking the MAPK/ERK pathway.

## Conclusions

In summary, our study first elucidated that RMZL exhibited protective effects in hepatic I/R injury. RMZL reduced hepatocyte damage by decreasing I/R-induced inflammatory responses. The activation of MAPK/ERK signaling in I/R enhanced inflammation and damage in hepatocytes. RMZL reduced hepatic I/R damage by inhibiting MAPK/ERK signaling.

### Electronic supplementary material

Below is the link to the electronic supplementary material.

Supplementary Material 1

## Data Availability

The datasets used or analyzed during this study can be made available from the corresponding author upon reasonable request.
