# Supplementary figures and images for: Remimazolam protects the liver from ischemia-reperfusion injury by inhibiting the MAPK/ERK pathway
Source: BMC Anesthesiol. 2024 Jul 25;24:251. doi: 10.1186/s12871-024-02641-3 (PMC11270846; doi:10.1186/s12871-024-02641-3)

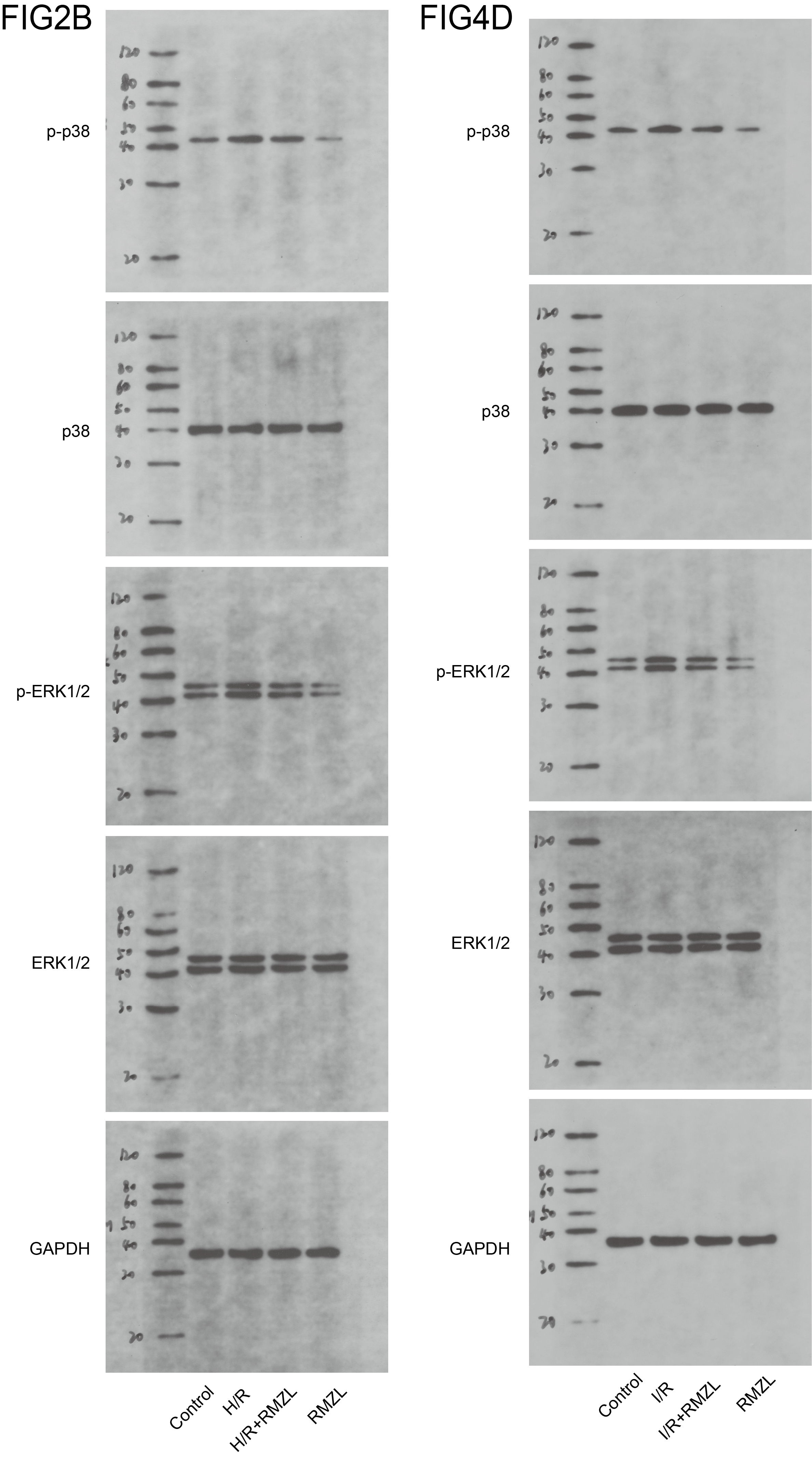

Supplement: Supplementary file 1 — Supplementary Material 1 [file 12871_2024_2641_MOESM1_ESM.tif]
